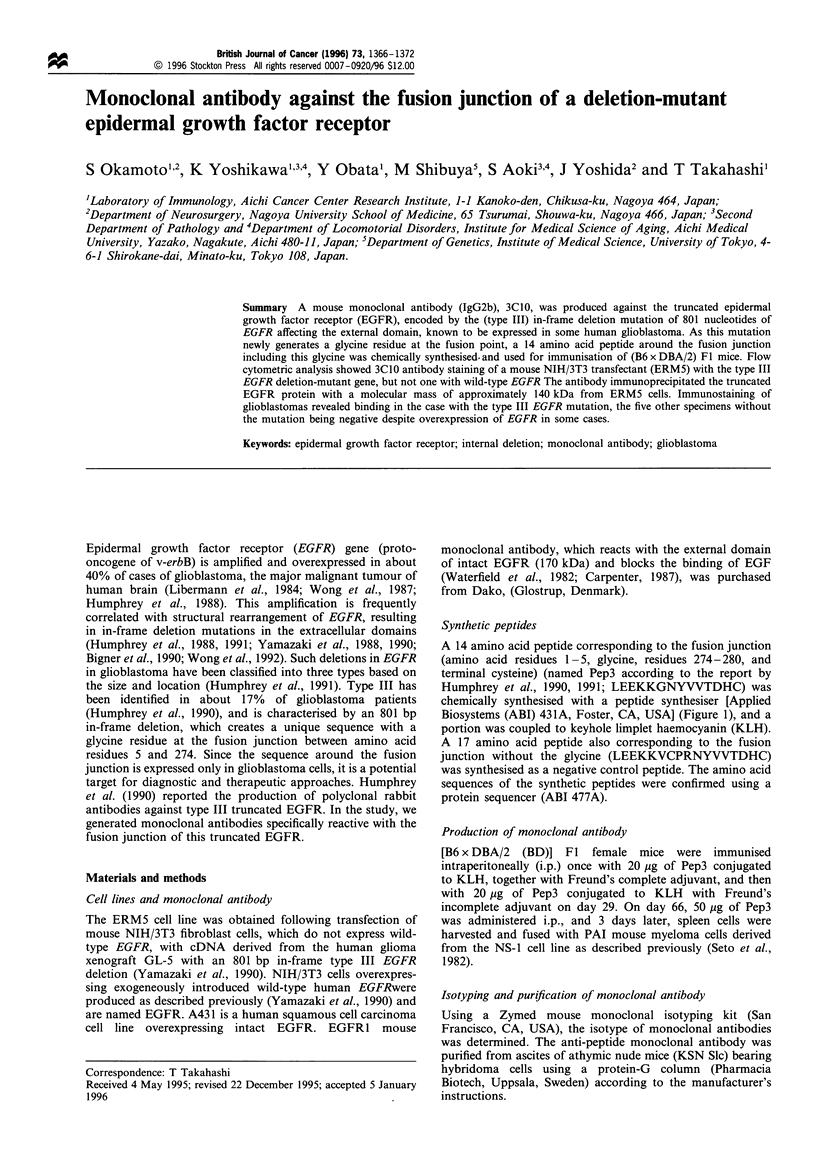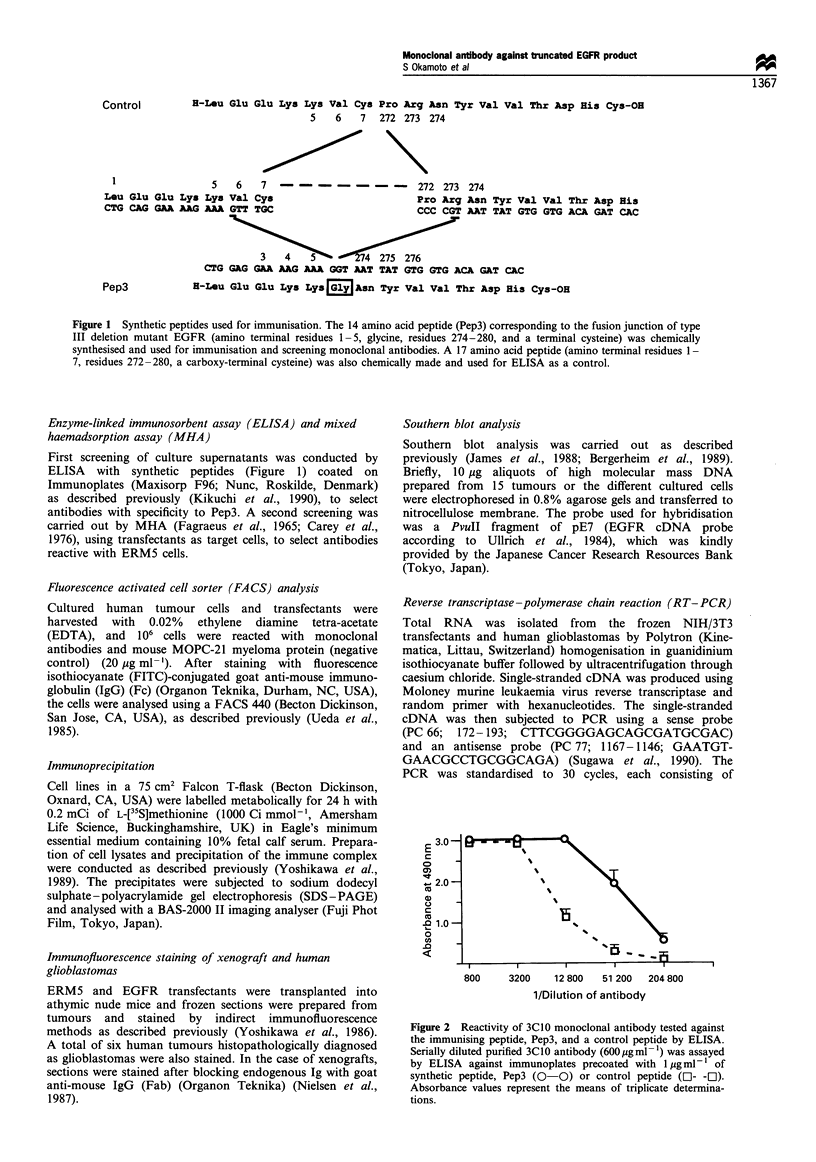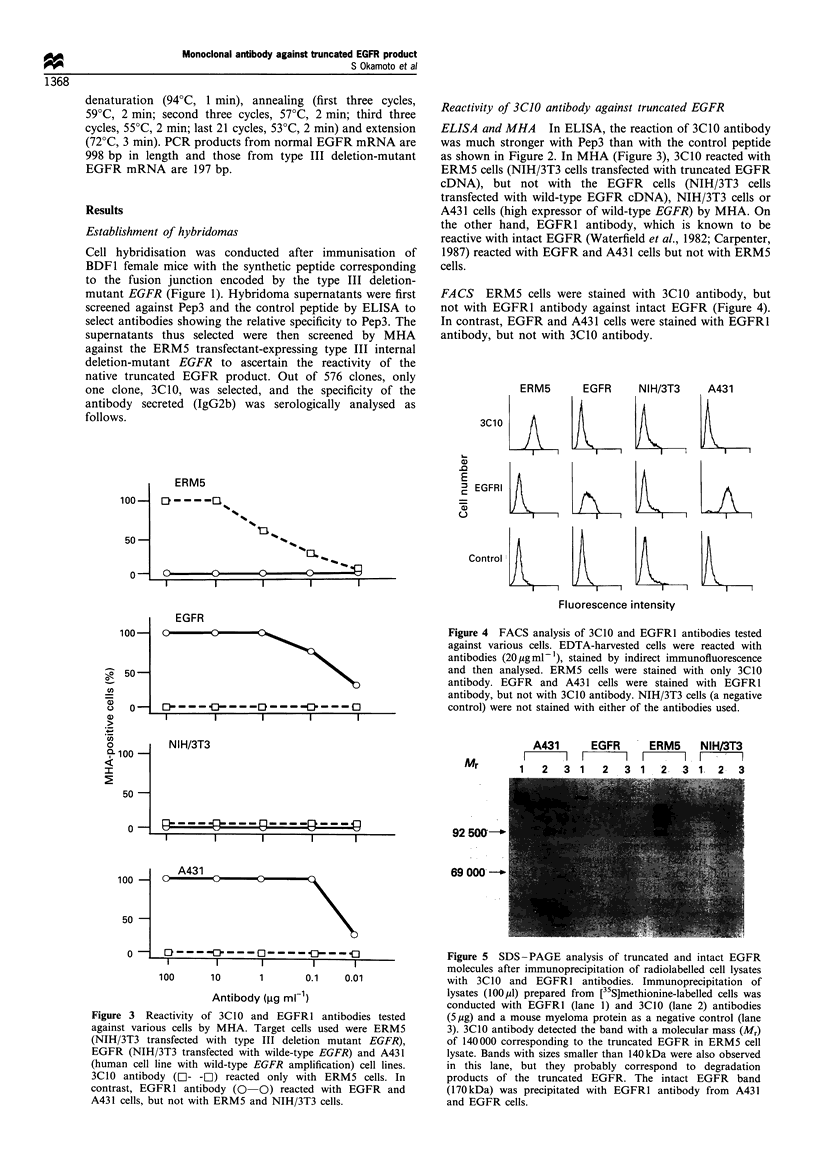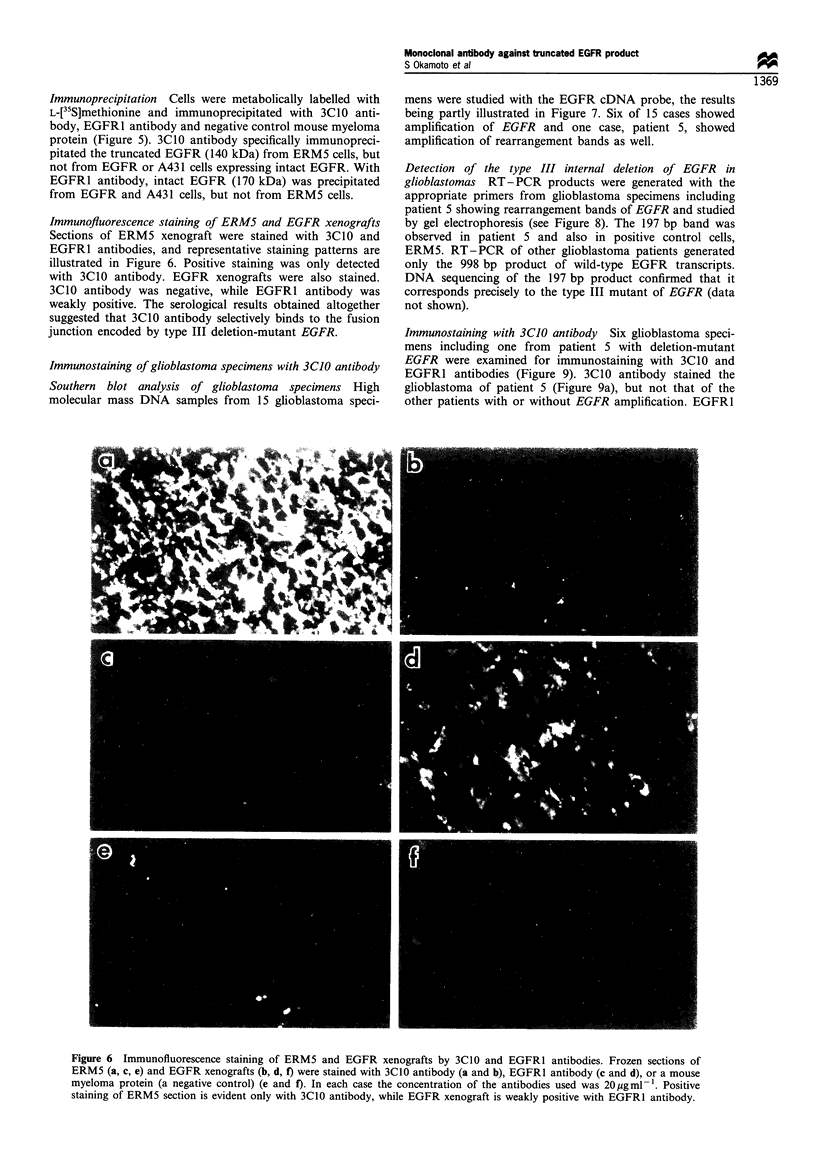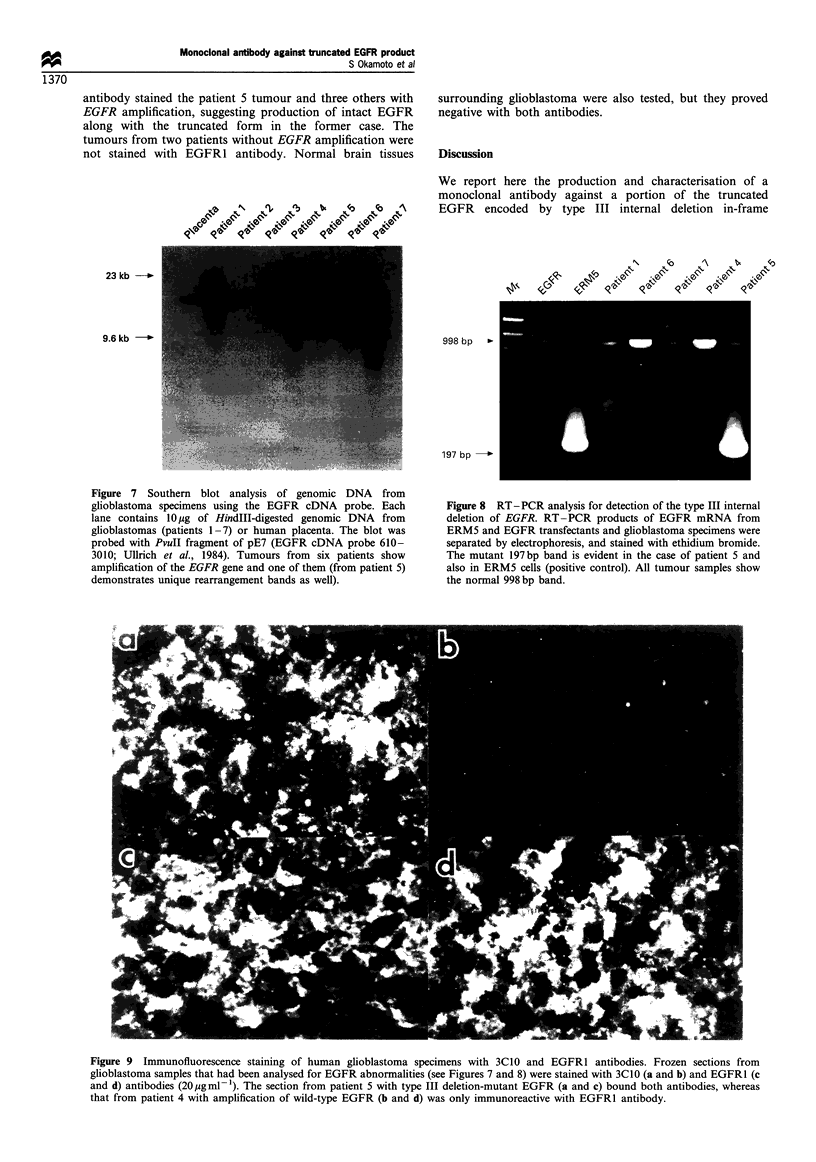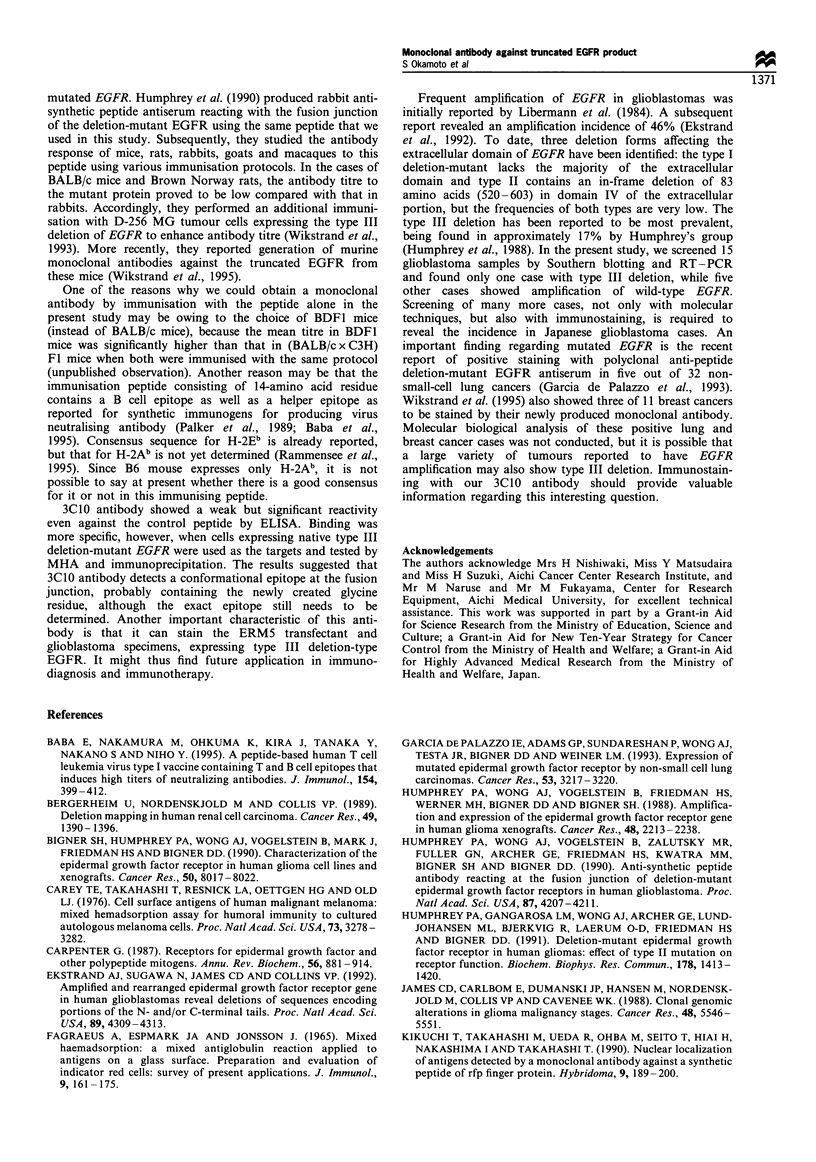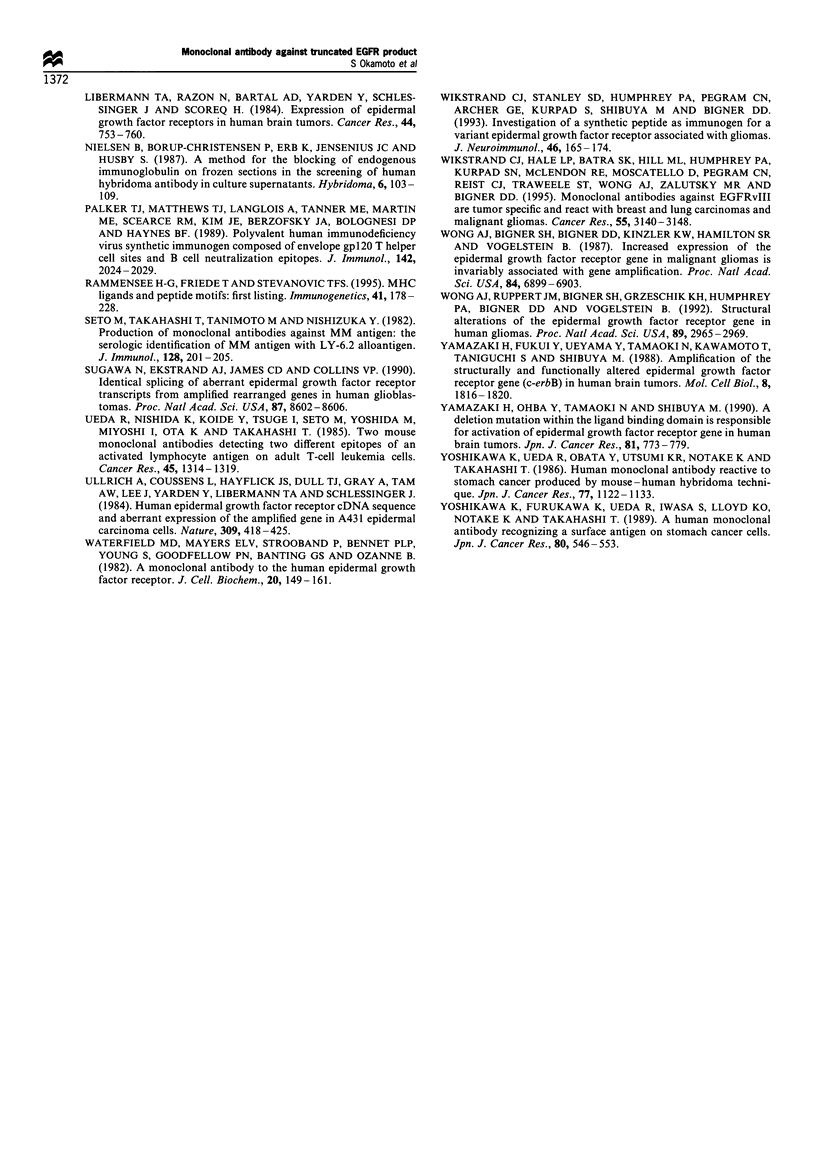# Monoclonal antibody against the fusion junction of a deletion-mutant epidermal growth factor receptor.

**DOI:** 10.1038/bjc.1996.260

**Published:** 1996-06

**Authors:** S. Okamoto, K. Yoshikawa, Y. Obata, M. Shibuya, S. Aoki, J. Yoshida, T. Takahashi

**Affiliations:** Laboratory of Immunology, Aichi Cancer Center Research Institute, Nagoya, Japan.

## Abstract

**Images:**


British Journal of Cancer (1996) 73, 1366-1372
© 1996 Stockton Press All rights reserved 0007-0920/96 $12.00

### Monoclonal antibody against the fusion junction of a deletion-mutant epidermal growth factor receptor

S Okamoto[1,2], K Yoshikawa[1,3,4], Y Obata[1], M Shibuya[5], S Aoki[3,4], J Yoshida[2] and T Takahashi[1]

*[1]Laboratory of Immunology, Aichi Cancer Center Research Institute, 1-1 Kanoko-den, Chikusa-ku, Nagoya 464, Japan; [2]Department of Neurosurgery, Nagoya University School of Medicine, 65 Tsurumai, Shouwa-ku, Nagoya 466, Japan; [3]Second Department of Pathology and [4]Department of Locomotorial Disorders, Institute for Medical Science of Aging, Aichi Medical University, Yazako, Nagakute, Aichi 480-11, Japan; [5]Department of Genetics, Institute of Medical Science, University of Tokyo, 4-6-1 Shirokane-dai, Minato-ku, Tokyo 108, Japan.*

**Summary** A mouse monoclonal antibody (IgG2b), 3C10, was produced against the truncated epidermal growth factor receptor (EGFR), encoded by the (type III) in-frame deletion mutation of 801 nucleotides of *EGFR* affecting the external domain, known to be expressed in some human glioblastoma. As this mutation newly generates a glycine residue at the fusion point, a 14 amino acid peptide around the fusion junction including this glycine was chemically synthesised and used for immunisation of (B6 × DBA/2) F1 mice. Flow cytometric analysis showed 3C10 antibody staining of a mouse NIH/3T3 transfectant (ERM5) with the type III *EGFR* deletion-mutant gene, but not one with wild-type *EGFR* The antibody immunoprecipitated the truncated EGFR protein with a molecular mass of approximately 140 kDa from ERM5 cells. Immunostaining of glioblastomas revealed binding in the case with the type III *EGFR* mutation, the five other specimens without the mutation being negative despite overexpression of *EGFR* in some cases.

**Keywords:** epidermal growth factor receptor; internal deletion; monoclonal antibody; glioblastoma

Epidermal growth factor receptor (*EGFR*) gene (proto-oncogene of v-*erb*B) is amplified and overexpressed in about 40% of cases of glioblastoma, the major malignant tumour of human brain (Libermann *et al.*, 1984; Wong *et al.*, 1987; Humphrey *et al.*, 1988). This amplification is frequently correlated with structural rearrangement of *EGFR*, resulting in in-frame deletion mutations in the extracellular domains (Humphrey *et al.*, 1988, 1991; Yamazaki *et al.*, 1988, 1990; Bigner *et al.*, 1990; Wong *et al.*, 1992). Such deletions in *EGFR* in glioblastoma have been classified into three types based on the size and location (Humphrey *et al.*, 1991). Type III has been identified in about 17% of glioblastoma patients (Humphrey *et al.*, 1990), and is characterised by an 801 bp in-frame deletion, which creates a unique sequence with a glycine residue at the fusion junction between amino acid residues 5 and 274. Since the sequence around the fusion junction is expressed only in glioblastoma cells, it is a potential target for diagnostic and therapeutic approaches. Humphrey *et al.* (1990) reported the production of polyclonal rabbit antibodies against type III truncated EGFR. In the study, we generated monoclonal antibodies specifically reactive with the fusion junction of this truncated EGFR.

### Materials and methods

### *Cell lines and monoclonal antibody*

The ERM5 cell line was obtained following transfection of mouse NIH/3T3 fibroblast cells, which do not express wild-type *EGFR*, with cDNA derived from the human glioma xenograft GL-5 with an 801 bp in-frame type III *EGFR* deletion (Yamazaki *et al.*, 1990). NIH/3T3 cells overexpressing exogeneously introduced wild-type human *EGFR*were produced as described previously (Yamazaki *et al.*, 1990) and are named EGFR. A431 is a human squamous cell carcinoma cell line overexpressing intact EGFR. EGFR1 mouse monoclonal antibody, which reacts with the external domain of intact EGFR (170 kDa) and blocks the binding of EGF (Waterfield *et al.*, 1982; Carpenter, 1987), was purchased from Dako, (Glostrup, Denmark).

### *Synthetic peptides*

A 14 amino acid peptide corresponding to the fusion junction (amino acid residues 1–5, glycine, residues 274–280, and terminal cysteine) (named Pep3 according to the report by Humphrey *et al.*, 1990, 1991; LEEKKGNYVVTDHC) was chemically synthesised with a peptide synthesiser [Applied Biosystems (ABI) 431A, Foster, CA, USA] (Figure 1), and a portion was coupled to keyhole limplet haemocyanin (KLH). A 17 amino acid peptide also corresponding to the fusion junction without the glycine (LEEKKVCPRNYVVTDHC) was synthesised as a negative control peptide. The amino acid sequences of the synthetic peptides were confirmed using a protein sequencer (ABI 477A).

### *Production of monoclonal antibody*

[B6 × DBA/2 (BD)] F1 female mice were immunised intraperitoneally (i.p.) once with 20 μg of Pep3 conjugated to KLH, together with Freund's complete adjuvant, and then with 20 μg of Pep3 conjugated to KLH with Freund's incomplete adjuvant on day 29. On day 66, 50 μg of Pep3 was administered i.p., and 3 days later, spleen cells were harvested and fused with PAI mouse myeloma cells derived from the NS-1 cell line as described previously (Seto *et al.*, 1982).

### *Isotyping and purification of monoclonal antibody*

Using a Zymed mouse monoclonal isotyping kit (San Francisco, CA, USA), the isotype of monoclonal antibodies was determined. The anti-peptide monoclonal antibody was purified from ascites of athymic nude mice (KSN Slc) bearing hybridoma cells using a protein-G column (Pharmacia Biotech, Uppsala, Sweden) according to the manufacturer's instructions.

Correspondence: T Takahashi
Received 4 May 1995; revised 22 December 1995; accepted 5 January 1996

**Figure 1** Synthetic peptides used for immunisation. The 14 amino acid peptide (Pep3) corresponding to the fusion junction of type III deletion mutant EGFR (amino terminal residues 1–5, glycine, residues 274–280, and a terminal cysteine) was chemically synthesised and used for immunisation and screening monoclonal antibodies. A 17 amino acid peptide (amino terminal residues 1–7, residues 272–280, a carboxy-terminal cysteine) was also chemically made and used for ELISA as a control.

*Enzyme-linked immunosorbent assay (ELISA) and mixed haemadsorption assay (MHA)*

First screening of culture supernatants was conducted by ELISA with synthetic peptides (Figure 1) coated on Immunoplates (Maxisorp F96; Nunc, Roskilde, Denmark) as described previously (Kikuchi *et al.*, 1990), to select antibodies with specificity to Pep3. A second screening was carried out by MHA (Fagraeus *et al.*, 1965; Carey *et al.*, 1976), using transfectants as target cells, to select antibodies reactive with ERM5 cells.

*Fluorescence activated cell sorter (FACS) analysis*

Cultured human tumour cells and transfectants were harvested with 0.02% ethylene diamine tetra-acetate (EDTA), and $10^6$ cells were reacted with monoclonal antibodies and mouse MOPC-21 myeloma protein (negative control) (20 $\mu$g ml$^{-1}$). After staining with fluorescence isothiocyanate (FITC)-conjugated goat anti-mouse immunoglobulin (IgG) (Fc) (Organon Teknika, Durham, NC, USA), the cells were analysed using a FACS 440 (Becton Dickinson, San Jose, CA, USA), as described previously (Ueda *et al.*, 1985).

*Immunoprecipitation*

Cell lines in a 75 cm$^2$ Falcon T-flask (Becton Dickinson, Oxnard, CA, USA) were labelled metabolically for 24 h with 0.2 mCi of L-[$^{35}$S]methionine (1000 Ci mmol$^{-1}$, Amersham Life Science, Buckinghamshire, UK) in Eagle's minimum essential medium containing 10% fetal calf serum. Preparation of cell lysates and precipitation of the immune complex were conducted as described previously (Yoshikawa *et al.*, 1989). The precipitates were subjected to sodium dodecyl sulphate–polyacrylamide gel electrophoresis (SDS–PAGE) and analysed with a BAS-2000 II imaging analyser (Fuji Phot Film, Tokyo, Japan).

*Immunofluorescence staining of xenograft and human glioblastomas*

ERM5 and EGFR transfectants were transplanted into athymic nude mice and frozen sections were prepared from tumours and stained by indirect immunofluorescence methods as described previously (Yoshikawa *et al.*, 1986). A total of six human tumours histopathologically diagnosed as glioblastomas were also stained. In the case of xenografts, sections were stained after blocking endogenous Ig with goat anti-mouse IgG (Fab) (Organon Teknika) (Nielsen *et al.*, 1987).

*Southern blot analysis*

Southern blot analysis was carried out as described previously (James *et al.*, 1988; Bergerheim *et al.*, 1989). Briefly, 10 $\mu$g aliquots of high molecular mass DNA prepared from 15 tumours or the different cultured cells were electrophoresed in 0.8% agarose gels and transferred to nitrocellulose membrane. The probe used for hybridisation was a *Pvu*II fragment of pE7 (EGFR cDNA probe according to Ullrich *et al.*, 1984), which was kindly provided by the Japanese Cancer Research Resources Bank (Tokyo, Japan).

*Reverse transcriptase–polymerase chain reaction (RT–PCR)*

Total RNA was isolated from the frozen NIH/3T3 transfectants and human glioblastomas by Polytron (Kinematica, Littau, Switzerland) homogenisation in guanidinium isothiocyanate buffer followed by ultracentrifugation through caesium chloride. Single-stranded cDNA was produced using Moloney murine leukaemia virus reverse transcriptase and random primer with hexanucleotides. The single-stranded cDNA was then subjected to PCR using a sense probe (PC 66; 172–193; CTTCGGGGAGCAGCGATGCGAC) and an antisense probe (PC 77; 1167–1146; GAATGT-GAACGCCTGCGGCAGA) (Sugawa *et al.*, 1990). The PCR was standardised to 30 cycles, each consisting of

**Figure 2** Reactivity of 3C10 monoclonal antibody tested against the immunising peptide, Pep3, and a control peptide by ELISA. Serially diluted purified 3C10 antibody (600 $\mu$g ml$^{-1}$) was assayed by ELISA against immunoplates precoated with 1 $\mu$g ml$^{-1}$ of synthetic peptide, Pep3 (○—○) or control peptide (□- -□). Absorbance values represent the means of triplicate determinations.

denaturation (94°C, 1 min), annealing (first three cycles, 59°C, 2 min; second three cycles, 57°C, 2 min; third three cycles, 55°C, 2 min; last 21 cycles, 53°C, 2 min) and extension (72°C, 3 min). PCR products from normal EGFR mRNA are 998 bp in length and those from type III deletion-mutant EGFR mRNA are 197 bp.

### Results

### *Establishment of hybridomas*

Cell hybridisation was conducted after immunisation of BDF1 female mice with the synthetic peptide corresponding to the fusion junction encoded by the type III deletion-mutant *EGFR* (Figure 1). Hybridoma supernatants were first screened against Pep3 and the control peptide by ELISA to select antibodies showing the relative specificity to Pep3. The supernatants thus selected were then screened by MHA against the ERM5 transfectant-expressing type III internal deletion-mutant *EGFR* to ascertain the reactivity of the native truncated EGFR product. Out of 576 clones, only one clone, 3C10, was selected, and the specificity of the antibody secreted (IgG2b) was serologically analysed as follows.

**Figure 3** Reactivity of 3C10 and EGFR1 antibodies tested against various cells by MHA. Target cells used were ERM5 (NIH/3T3 transfected with type III deletion mutant *EGFR*), EGFR (NIH/3T3 transfected with wilde-type *EGFR*) and A431 (human cell line with wild-type *EGFR* amplification) cell lines. 3C10 antibody (□- -□) reacted only with ERM5 cells. In contrast, EGFR1 antibody (O—O) reacted with EGFR and A431 cells, but not with ERM5 and NIH/3T3 cells.

### *Reactivity of 3C10 antibody against truncated EGFR*

*ELISA and MHA* In ELISA, the reaction of 3C10 antibody was much stronger with Pep3 than with the control peptide as shown in Figure 2. In MHA (Figure 3), 3C10 reacted with ERM5 cells (NIH/3T3 cells transfected with truncated EGFR cDNA), but not with the EGFR cells (NIH/3T3 cells transfected with wild-type EGFR cDNA), NIH/3T3 cells or A431 cells (high expressor of wild-type *EGFR*) by MHA. On the other hand, EGFR1 antibody, which is known to be reactive with intact EGFR (Waterfield *et al*., 1982; Carpenter, 1987) reacted with EGFR and A431 cells but not with ERM5 cells.

*FACS* ERM5 cells were stained with 3C10 antibody, but not with EGFR1 antibody against intact EGFR (Figure 4). In contrast, EGFR and A431 cells were stained with EGFR1 antibody, but not with 3C10 antibody.

**Figure 4** FACS analysis of 3C10 and EGFR1 antibodies tested against various cells. EDTA-harvested cells were reacted with antibodies (20 $\mu$g ml$^{-1}$), stained by indirect immunofluorescence and then analysed. ERM5 cells were stained with only 3C10 antibody. EGFR and A431 cells were stained with EGFR1 antibody, but not with 3C10 antibody. NIH/3T3 cells (a negative control) were not stained with either of the antibodies used.

**Figure 5** SDS-PAGE analysis of truncated and intact EGFR molecules after immunoprecipitation of radiolabelled cell lysates with 3C10 and EGFR1 antibodies. Immunoprecipitation of lysates (100 $\mu$l) prepared from [$^{35}$S]methionine-labelled cells was conducted with EGFR1 (lane 1) and 3C10 (lane 2) antibodies (5 $\mu$g) and a mouse myeloma protein as a negative control (lane 3). 3C10 antibody detected the band with a molecular mass ($M_r$) of 140 000 corresponding to the truncated EGFR in ERM5 cell lysate. Bands with sizes smaller than 140 kDa were also observed in this lane, but they probably correspond to degradation products of the truncated EGFR. The intact EGFR band (170 kDa) was precipitated with EGFR1 antibody from A431 and EGFR cells.

*Immunoprecipitation* Cells were metabolically labelled with L-[$^{35}$S]methionine and immunoprecipitated with 3C10 antibody, EGFR1 antibody and negative control mouse myeloma protein (Figure 5). 3C10 antibody specifically immunoprecipitated the truncated EGFR (140 kDa) from ERM5 cells, but not from EGFR or A431 cells expressing intact EGFR. With EGFR1 antibody, intact EGFR (170 kDa) was precipitated from EGFR and A431 cells, but not from ERM5 cells.

*Immunofluorescence staining of ERM5 and EGFR xenografts* Sections of ERM5 xenograft were stained with 3C10 and EGFR1 antibodies, and representative staining patterns are illustrated in Figure 6. Positive staining was only detected with 3C10 antibody. EGFR xenografts were also stained. 3C10 antibody was negative, while EGFR1 antibody was weakly positive. The serological results obtained altogether suggested that 3C10 antibody selectively binds to the fusion junction encoded by type III deletion-mutant *EGFR*.

*Immunostaining of glioblastoma specimens with 3C10 antibody*

*Southern blot analysis of glioblastoma specimens* High molecular mass DNA samples from 15 glioblastoma specimens were studied with the EGFR cDNA probe, the results being partly illustrated in Figure 7. Six of 15 cases showed amplification of *EGFR* and one case, patient 5, showed amplification of rearrangement bands as well.

*Detection of the type III internal deletion of EGFR in glioblastomas* RT-PCR products were generated with the appropriate primers from glioblastoma specimens including patient 5 showing rearrangement bands of *EGFR* and studied by gel electrophoresis (see Figure 8). The 197 bp band was observed in patient 5 and also in positive control cells, ERM5. RT-PCR of other glioblastoma patients generated only the 998 bp product of wild-type EGFR transcripts. DNA sequencing of the 197 bp product confirmed that it corresponds precisely to the type III mutant of *EGFR* (data not shown).

*Immunostaining with 3C10 antibody* Six glioblastoma specimens including one from patient 5 with deletion-mutant *EGFR* were examined for immunostaining with 3C10 and EGFR1 antibodies (Figure 9). 3C10 antibody stained the glioblastoma of patient 5 (Figure 9a), but not that of the other patients with or without *EGFR* amplification. EGFR1

**Figure 6** Immunofluorescence staining of ERM5 and EGFR xenografts by 3C10 and EGFR1 antibodies. Frozen sections of ERM5 (**a**, **c**, **e**) and EGFR xenografts (**b**, **d**, **f**) were stained with 3C10 antibody (**a** and **b**), EGFR1 antibody (**c** and **d**), or a mouse myeloma protein (a negative control) (**e** and **f**). In each case the concentration of the antibodies used was 20 $\mu$g ml$^{-1}$. Positive staining of ERM5 section is evident only with 3C10 antibody, while EGFR xenograft is weakly positive with EGFR1 antibody.

antibody stained the patient 5 tumour and three others with *EGFR* amplification, suggesting production of intact EGFR along with the truncated form in the former case. The tumours from two patients without *EGFR* amplification were not stained with EGFR1 antibody. Normal brain tissues surrounding glioblastoma were also tested, but they proved negative with both antibodies.

**Figure 7** Southern blot analysis of genomic DNA from glioblastoma specimens using the EGFR cDNA probe. Each lane contains 10 $\mu$g of *Hin*dIII-digested genomic DNA from glioblastomas (patients 1–7) or human placenta. The blot was probed with *Pvu*II fragment of pE7 (EGFR cDNA probe 610–3010; Ullrich *et al.*, 1984). Tumours from six patients show amplification of the *EGFR* gene and one of them (from patient 5) demonstrates unique rearrangement bands as well).

### Discussion

We report here the production and characterisation of a monoclonal antibody against a portion of the truncated EGFR encoded by type III internal deletion in-frame

**Figure 8** RT–PCR analysis for detection of the type III internal deletion of *EGFR*. RT–PCR products of EGFR mRNA from ERM5 and EGFR transfectants and glioblastoma specimens were separated by electrophoresis, and stained with ethidium bromide. The mutant 197 bp band is evident in the case of patient 5 and also in ERM5 cells (positive control). All tumour samples show the normal 998 bp band.

**Figure 9** Immunofluorescence staining of human glioblastoma specimens with 3C10 and EGFR1 antibodies. Frozen sections from glioblastoma samples that had been analysed for EGFR abnormalities (see Figures 7 and 8) were stained with 3C10 (**a** and **b**) and EGFR1 (**c** and **d**) antibodies (20 $\mu g\,ml^{-1}$). The section from patient 5 with type III deletion-mutant EGFR (**a** and **c**) bound both antibodies, whereas that from patient 4 with amplification of wild-type EGFR (**b** and **d**) was only immunoreactive with EGFR1 antibody.

mutated *EGFR*. Humphrey *et al.* (1990) produced rabbit anti-synthetic peptide antiserum reacting with the fusion junction of the deletion-mutant EGFR using the same peptide that we used in this study. Subsequently, they studied the antibody response of mice, rats, rabbits, goats and macaques to this peptide using various immunisation protocols. In the cases of BALB/c mice and Brown Norway rats, the antibody titre to the mutant protein proved to be low compared with that in rabbits. Accordingly, they performed an additional immunisation with D-256 MG tumour cells expressing the type III deletion of *EGFR* to enhance antibody titre (Wikstrand *et al.*, 1993). More recently, they reported generation of murine monoclonal antibodies against the truncated EGFR from these mice (Wikstrand *et al.*, 1995).

One of the reasons why we could obtain a monoclonal antibody by immunisation with the peptide alone in the present study may be owing to the choice of BDF1 mice (instead of BALB/c mice), because the mean titre in BDF1 mice was significantly higher than that in (BALB/c $\times$ C3H) F1 mice when both were immunised with the same protocol (unpublished observation). Another reason may be that the immunisation peptide consisting of 14-amino acid residue contains a B cell epitope as well as a helper epitope as reported for synthetic immunogens for producing virus neutralising antibody (Palker *et al.*, 1989; Baba *et al.*, 1995). Consensus sequence for H-2E$^b$ is already reported, but that for H-2A$^b$ is not yet determined (Rammensee *et al.*, 1995). Since B6 mouse expresses only H-2A$^b$, it is not possible to say at present whether there is a good consensus for it or not in this immunising peptide.

3C10 antibody showed a weak but significant reactivity even against the control peptide by ELISA. Binding was more specific, however, when cells expressing native type III deletion-mutant *EGFR* were used as the targets and tested by MHA and immunoprecipitation. The results suggested that 3C10 antibody detects a conformational epitope at the fusion junction, probably containing the newly created glycine residue, although the exact epitope still needs to be determined. Another important characteristic of this antibody is that it can stain the ERM5 transfectant and glioblastoma specimens, expressing type III deletion-type EGFR. It might thus find future application in immunodiagnosis and immunotherapy.

Frequent amplification of *EGFR* in glioblastomas was initially reported by Libermann *et al.* (1984). A subsequent report revealed an amplification incidence of 46% (Ekstrand *et al.*, 1992). To date, three deletion forms affecting the extracellular domain of *EGFR* have been identified: the type I deletion-mutant lacks the majority of the extracellular domain and type II contains an in-frame deletion of 83 amino acids (520–603) in domain IV of the extracellular portion, but the frequencies of both types are very low. The type III deletion has been reported to be most prevalent, being found in approximately 17% by Humphrey's group (Humphrey *et al.*, 1988). In the present study, we screened 15 glioblastoma samples by Southern blotting and RT–PCR and found only one case with type III deletion, while five other cases showed amplification of wild-type *EGFR*. Screening of many more cases, not only with molecular techniques, but also with immunostaining, is required to reveal the incidence in Japanese glioblastoma cases. An important finding regarding mutated *EGFR* is the recent report of positive staining with polyclonal anti-peptide deletion-mutant EGFR antiserum in five out of 32 non-small-cell lung cancers (Garcia de Palazzo *et al.*, 1993). Wikstrand *et al.* (1995) also showed three of 11 breast cancers to be stained by their newly produced monoclonal antibody. Molecular biological analysis of these positive lung and breast cancer cases was not conducted, but it is possible that a large variety of tumours reported to have *EGFR* amplification may also show type III deletion. Immunostaining with our 3C10 antibody should provide valuable information regarding this interesting question.

**Acknowledgements**
The authors acknowledge Mrs H Nishiwaki, Miss Y Matsudaira and Miss H Suzuki, Aichi Cancer Center Research Institute, and Mr M Naruse and Mr M Fukayama, Center for Research Equipment, Aichi Medical University, for excellent technical assistance. This work was supported in part by a Grant-in Aid for Science Research from the Ministry of Education, Science and Culture; a Grant-in Aid for New Ten-Year Strategy for Cancer Control from the Ministry of Health and Welfare; a Grant-in Aid for Highly Advanced Medical Research from the Ministry of Health and Welfare, Japan.

### References

BABA E, NAKAMURA M, OHKUMA K, KIRA J, TANAKA Y, NAKANO S AND NIHO Y. (1995). A peptide-based human T cell leukemia virus type I vaccine containing T and B cell epitopes that induces high titers of neutralizing antibodies. *J. Immunol.*, **154**, 399–412.

BERGERHEIM U, NORDENSKJOLD M AND COLLIS VP. (1989). Deletion mapping in human renal cell carcinoma. *Cancer Res.*, **49**, 1390–1396.

BIGNER SH, HUMPHREY PA, WONG AJ, VOGELSTEIN B, MARK J, FRIEDMAN HS AND BIGNER DD. (1990). Characterization of the epidermal growth factor receptor in human glioma cell lines and xenografts. *Cancer Res.*, **50**, 8017–8022.

CAREY TE, TAKAHASHI T, RESNICK LA, OETTGEN HG AND OLD LJ. (1976). Cell surface antigens of human malignant melanoma: mixed hemadsorption assay for humoral immunity to cultured autologous melanoma cells. *Proc. Natl Acad. Sci. USA*, **73**, 3278–3282.

CARPENTER G. (1987). Receptors for epidermal growth factor and other polypeptide mitogens. *Annu. Rev. Biochem.*, **56**, 881–914.

EKSTRAND AJ, SUGAWA N, JAMES CD AND COLLINS VP. (1992). Amplified and rearranged epidermal growth factor receptor gene in human glioblastomas reveal deletions of sequences encoding portions of the N- and/or C-terminal tails. *Proc. Natl Acad. Sci. USA*, **89**, 4309–4313.

FAGRAEUS A, ESPMARK JA AND JONSSON J. (1965). Mixed haemadsorption: a mixed antiglobulin reaction applied to antigens on a glass surface. Preparation and evaluation of indicator red cells: survey of present applications. *J. Immunol.*, **9**, 161–175.

GARCIA DE PALAZZO IE, ADAMS GP, SUNDARESHAN P, WONG AJ, TESTA JR, BIGNER DD AND WEINER LM. (1993). Expression of mutated epidermal growth factor receptor by non-small cell lung carcinomas. *Cancer Res.*, **53**, 3217–3220.

HUMPHREY PA, WONG AJ, VOGELSTEIN B, FRIEDMAN HS, WERNER MH, BIGNER DD AND BIGNER SH. (1988). Amplification and expression of the epidermal growth factor receptor gene in human glioma xenografts. *Cancer Res.*, **48**, 2213–2238.

HUMPHREY PA, WONG AJ, VOGELSTEIN B, ZALUTSKY MR, FULLER GN, ARCHER GE, FRIEDMAN HS, KWATRA MM, BIGNER SH AND BIGNER DD. (1990). Anti-synthetic peptide antibody reacting at the fusion junction of deletion-mutant epidermal growth factor receptors in human glioblastoma. *Proc. Natl Acad. Sci. USA*, **87**, 4207–4211.

HUMPHREY PA, GANGAROSA LM, WONG AJ, ARCHER GE, LUND-JOHANSEN ML, BJERKVIG R, LAERUM O-D, FRIEDMAN HS AND BIGNER DD. (1991). Deletion-mutant epidermal growth factor receptor in human gliomas: effect of type II mutation on receptor function. *Biochem. Biophys. Res. Commun.*, **178**, 1413–1420.

JAMES CD, CARLBOM E, DUMANSKI JP, HANSEN M, NORDENSKJOLD M, COLLIS VP AND CAVENEE WK. (1988). Clonal genomic alterations in glioma malignancy stages. *Cancer Res.*, **48**, 5546–5551.

KIKUCHI T, TAKAHASHI M, UEDA R, OHBA M, SEITO T, HIAI H, NAKASHIMA I AND TAKAHASHI T. (1990). Nuclear localization of antigens detected by a monoclonal antibody against a synthetic peptide of rfp finger protein. *Hybridoma*, **9**, 189–200.

LIBERMANN TA, RAZON N, BARTAL AD, YARDEN Y, SCHLESSINGER J AND SCOREQ H. (1984). Expression of epidermal growth factor receptors in human brain tumors. *Cancer Res.*, **44,** 753–760.

NIELSEN B, BORUP-CHRISTENSEN P, ERB K, JENSENIUS JC AND HUSBY S. (1987). A method for the blocking of endogenous immunoglobulin on frozen sections in the screening of human hybridoma antibody in culture supernatants. *Hybridoma*, **6,** 103–109.

PALKER TJ, MATTHEWS TJ, LANGLOIS A, TANNER ME, MARTIN ME, SCEARCE RM, KIM JE, BERZOFSKY JA, BOLOGNESI DP AND HAYNES BF. (1989). Polyvalent human immunodeficiency virus synthetic immunogen composed of envelope gp120 T helper cell sites and B cell neutralization epitopes. *J. Immunol.*, **142,** 2024–2029.

RAMMENSEE H-G, FRIEDE T AND STEVANOVIC TFS. (1995). MHC ligands and peptide motifs: first listing. *Immunogenetics*, **41,** 178–228.

SETO M, TAKAHASHI T, TANIMOTO M AND NISHIZUKA Y. (1982). Production of monoclonal antibodies against MM antigen: the serologic identification of MM antigen with LY-6.2 alloantigen. *J. Immunol.*, **128,** 201–205.

SUGAWA N, EKSTRAND AJ, JAMES CD AND COLLINS VP. (1990). Identical splicing of aberrant epidermal growth factor receptor transcripts from amplified rearranged genes in human glioblastomas. *Proc. Natl Acad. Sci. USA*, **87,** 8602–8606.

UEDA R, NISHIDA K, KOIDE Y, TSUGE I, SETO M, YOSHIDA M, MIYOSHI I, OTA K AND TAKAHASHI T. (1985). Two mouse monoclonal antibodies detecting two different epitopes of an activated lymphocyte antigen on adult T-cell leukemia cells. *Cancer Res.*, **45,** 1314–1319.

ULLRICH A, COUSSENS L, HAYFLICK JS, DULL TJ, GRAY A, TAM AW, LEE J, YARDEN Y, LIBERMANN TA AND SCHLESSINGER J. (1984). Human epidermal growth factor receptor cDNA sequence and aberrant expression of the amplified gene in A431 epidermal carcinoma cells. *Nature*, **309,** 418–425.

WATERFIELD MD, MAYERS ELV, STROOBAND P, BENNET PLP, YOUNG S, GOODFELLOW PN, BANTING GS AND OZANNE B. (1982). A monoclonal antibody to the human epidermal growth factor receptor. *J. Cell. Biochem.*, **20,** 149–161.

WIKSTRAND CJ, STANLEY SD, HUMPHREY PA, PEGRAM CN, ARCHER GE, KURPAD S, SHIBUYA M AND BIGNER DD. (1993). Investigation of a synthetic peptide as immunogen for a variant epidermal growth factor receptor associated with gliomas. *J. Neuroimmunol.*, **46,** 165–174.

WIKSTRAND CJ, HALE LP, BATRA SK, HILL ML, HUMPHREY PA, KURPAD SN, MCLENDON RE, MOSCATELLO D, PEGRAM CN, REIST CJ, TRAWEELE ST, WONG AJ, ZALUTSKY MR AND BIGNER DD. (1995). Monoclonal antibodies against EGFRvIII are tumor specific and react with breast and lung carcinomas and malignant gliomas. *Cancer Res.*, **55,** 3140–3148.

WONG AJ, BIGNER SH, BIGNER DD, KINZLER KW, HAMILTON SR AND VOGELSTEIN B. (1987). Increased expression of the epidermal growth factor receptor gene in malignant gliomas is invariably associated with gene amplification. *Proc. Natl Acad. Sci. USA*, **84,** 6899–6903.

WONG AJ, RUPPERT JM, BIGNER SH, GRZESCHIK KH, HUMPHREY PA, BIGNER DD AND VOGELSTEIN B. (1992). Structural alterations of the epidermal growth factor receptor gene in human gliomas. *Proc. Natl Acad. Sci. USA*, **89,** 2965–2969.

YAMAZAKI H, FUKUI Y, UEYAMA Y, TAMAOKI N, KAWAMOTO T, TANIGUCHI S AND SHIBUYA M. (1988). Amplification of the structurally and functionally altered epidermal growth factor receptor gene (c-*erb*B) in human brain tumors. *Mol. Cell Biol.*, **8,** 1816–1820.

YAMAZAKI H, OHBA Y, TAMAOKI N AND SHIBUYA M. (1990). A deletion mutation within the ligand binding domain is responsible for activation of epidermal growth factor receptor gene in human brain tumors. *Jpn. J. Cancer Res.*, **81,** 773–779.

YOSHIKAWA K, UEDA R, OBATA Y, UTSUMI KR, NOTAKE K AND TAKAHASHI T. (1986). Human monoclonal antibody reactive to stomach cancer produced by mouse–human hybridoma technique. *Jpn. J. Cancer Res.*, **77,** 1122–1133.

YOSHIKAWA K, FURUKAWA K, UEDA R, IWASA S, LLOYD KO, NOTAKE K AND TAKAHASHI T. (1989). A human monoclonal antibody recognizing a surface antigen on stomach cancer cells. *Jpn. J. Cancer Res.*, **80,** 546–553.